# Inhibition of FGF‐FGFR and VEGF‐VEGFR signalling in cancer treatment

**DOI:** 10.1111/cpr.13009

**Published:** 2021-03-02

**Authors:** Guihong Liu, Tao Chen, Zhenyu Ding, Yang Wang, Yuquan Wei, Xiawei Wei

**Affiliations:** ^1^ Laboratory of Aging Research and Cancer Drug Target State Key Laboratory of Biotherapy National Clinical Research Center for Geriatrics West China Hospital Sichuan University Chengdu China; ^2^ Cardiology Department Chengdu NO.7 People’s Hospital Chengdu Tumor Hospital Chengdu China; ^3^ Department of Biotherapy State Key Laboratory of Biotherapy Cancer Center West China Hospital Sichuan University Chengdu China

**Keywords:** FGFR/VEGFR inhibitor, fibroblast growth factor, fibroblast growth factor receptor, vascular endothelial growth factor, vascular endothelial growth factor receptor

## Abstract

The sites of targeted therapy are limited and need to be expanded. The FGF‐FGFR signalling plays pivotal roles in the oncogenic process, and FGF/FGFR inhibitors are a promising method to treat FGFR‐altered tumours. The VEGF‐VEGFR signalling is the most crucial pathway to induce angiogenesis, and inhibiting this cascade has already got success in treating tumours. While both their efficacy and antitumour spectrum are limited, combining FGF/FGFR inhibitors with VEGF/VEGFR inhibitors are an excellent way to optimize the curative effect and expand the antitumour range because their combination can target both tumour cells and the tumour microenvironment. In addition, biomarkers need to be developed to predict the efficacy, and combination with immune checkpoint inhibitors is a promising direction in the future. The article will discuss the FGF‐FGFR signalling pathway, the VEGF‐VEGFR signalling pathway, the rationale of combining these two signalling pathways and recent small‐molecule FGFR/VEGFR inhibitors based on clinical trials.

## Graphical abstract

Targeted therapies interfering with oncogenic driver alterations have achieved remarkable success in limited types of cancer with certain driver gene alterations (*Nat Rev Clin Oncol*, 2017; *Lancet Oncol*, 2018; *Jama*, 2019; *Lancet*, 2017). Novel therapeutics targeting other cancer driver alterations are urgently needed to be developed to improve the life quantity of the patients and prolong their life span. The FGF‐FGFR signalling plays pivotal roles in both the physiological and oncogenic processes (*Nat Rev Clin Oncol*, 2019), but FGFRs are constitutively active in malignant cells because of the upregulation of FGF and FGFR genetic alterations (*Nat Rev Clin Oncol*, 2019). Targeting FGF‐FGFR signalling is a promising method to treat FGFR‐altered tumours (*New Engl J Med*, 2019; *Lancet Oncol*, 2020), but patients receive limited effects by targeting only the FGF‐FGFR pathway in most clinical practice (*Nat Rev Cancer*, 2017; *Eur J Med Chem*, 2020). VEGF‐VEGFR signalling pathway also attracts our attention. The growth of tumours relies on blood supply and VEGFs are proved to be the most important angiogenic factors (*Nat Rev Drug Discov*, 2016). Accordingly, inhibition of the VEGF‐VEGFR signalling pathway is believed to suppress tumour development (*New Engl J Med*, 1971). Here we propose the simultaneous inhibition of the FGF‐FGFR pathway and VEGF‐VEGFR pathway. In terms of mechanism, the combination can target tumour cells and tumour microenvironment at the same time (*Clin Cancer Res*, 2019). FGFR/VEGFR inhibitors have better effects and broaden the indications in clinical use (*Nat Commun*, 2020; *JAMA Oncol*, 2018; *The Lancet Oncology*, 2020).

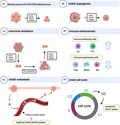

## 1 INTRODUCTION

Targeted therapies interfering with oncogenic driver alterations have achieved great success in chronic myeloid leukaemia (CML) with BCR‐ABL fusions,^1^ melanoma with BRAF V600E mutations,^2^ lung cancer with EGFR mutations^3^ and breast cancer with HER2 amplification.^4^ However, approved targeted agents can only block limited types of cancer with specific driver gene alterations. The development of novel therapeutics targeting other cancer driver alterations is extremely urgent to improve patients’ prognosis.

The fibroblast growth factor (FGF)‐FGF receptor (FGFR) signalling cascade plays a pivotal role in driving cancer growth. Anti‐FGF or FGFR therapy is a promising way to treat tumours with FGF and (or) FGFR alterations.^5^ With the accelerated approval of erdafitinib for FGFR‐altered urothelial carcinoma in April 2019 and pemigatinib for cholangiocarcinoma with FGFR2 fusion or other rearrangements in April 2020,^6^, ^7^ the FGF‐FGFR signalling pathway has received more attention. However, patients often received limited clinical benefits in treatment with agents that only block the FGF‐FGFR signalling cascade.^5^ Combination of the inhibitory of the FGF‐FGFR signalling pathway with other mechanisms is a promising way to solve this puzzle.

Tumours growth relies on blood supply, and vascular endothelial growth factors (VEGFs) are essential angiogenesis stimulators.^8^ Through inhibiting the VEGF‐VEGF receptor (VEGFR) signalling, anti‐VEGF or VEGR agents have been approved for use in various solid tumours, but they lead only to mild clinical benefits in most situations.^9^

Herein, in this review, we mainly focus on the FGF‐FGFR signalling pathway, the VEGF‐VEGFR signalling pathway, the rationale of combining these two pathways and recent small‐molecule FGFR/VEGFR inhibitors based on clinical trials.

## 2 FGF‐FGFR SIGNALLING

### 2.1 FGFs

Fibroblast growth factor was first extracted from bovine pituitary in 1973, partially purified in 1975, and finally purified to homogeneity in 1983.^10^, ^11^, ^12^ The mammalian FGF family comprises 22 members, including FGF1‐FGF23. Human FGF19 and mouse FGF15 are analogs. Phylogenetic and gene locus analyses divide the FGF family into seven subfamilies. Their action mechanisms classify these subfamilies into three groups, the canonical FGF subfamily including the FGF1/2/5, FGF3/4/6, FGF7/10/22, FGF8/17/18 and FGF9/16/20 subfamilies, the endocrine FGF19/21/23 subfamily and the intracellular FGF11/12/13/14 subfamily.^13^, ^14^

### 2.2 FGFRs

The canonical and endocrine FGFs produce their biological actions by signalling through FGFRs (FGFR1‐4), which are expressed on the cell membrane, consisted of three extracellular immunoglobulin (Ig)‐like domains (I, II, III), a transmembrane domain (TM) and two intracellular tyrosine kinase domains (TK1 and TK2).^15^, ^16^ FGFR1‐3 generate two additional major splice variants of Ig‐like domain III, referred to as IIIb and IIIc, concerned with ligand‐binding specificity. In contrast to other family members, FGFR4 has only one isoform.^17^ The FGF‐binding pocket is formed by the II and III subregions.^18^ The FGFR TK domains are the heart of the action, responsible for offering ATP‐binding area and phosphorylating tyrosine residues to gradually increase catalytic activity tens to thousands of times. Finally, the specific phosphorylation site can bind and phosphorylate substrate proteins to activate multiple signal transduction pathways.^19^ Take FGFR1 as an example; seven phosphorylatable tyrosine residues have been identified, that is, Y463, Y583, Y585, Y653, Y654, Y730 and Y766.^20^ Among these, Y653 and Y654 are essential for kinase activity, and phospho‐Y766 serves as a binding site for downstream protein.^21^ There are several critical functional loops in the intracellular domain, one of which is an activation loop (A‐loop). The conformation of the highly conserved Asp‐Phe‐Gly motif (DFG‐motif) in the A‐loop is an indicator of kinase activity status. The DFG‐motif exists in two states: the active DFG‐in and inactive DFG‐out conformations, relating to the mechanism of FGFR inhibitors, which we will describe more below.^22^

### 2.3 Extracellular FGF associated cofactors

Heparin and heparan sulphate proteoglycans (HSPG) act as essential cofactors for the binding of canonical FGFs.^23^ Unlike the canonical FGFs, endocrine FGFs require Klotho co‐receptors instead to act as cofactors for FGFR activation. αKlotho is a cofactor for FGF23 and βKlotho for FGF15/19 and FGF21.^24^ All cofactors are single‐pass TM proteins, binding to extracellular Ig‐like domain II of FGFR. This 1:1:1 FGF‐HS/Klotho‐FGFR ternary complex structure leads to conformational changes that stabilize a symmetric 2:2:2dimer.^25^

### 2.4 Intracellular signal transduction

**FIGURE 1:**
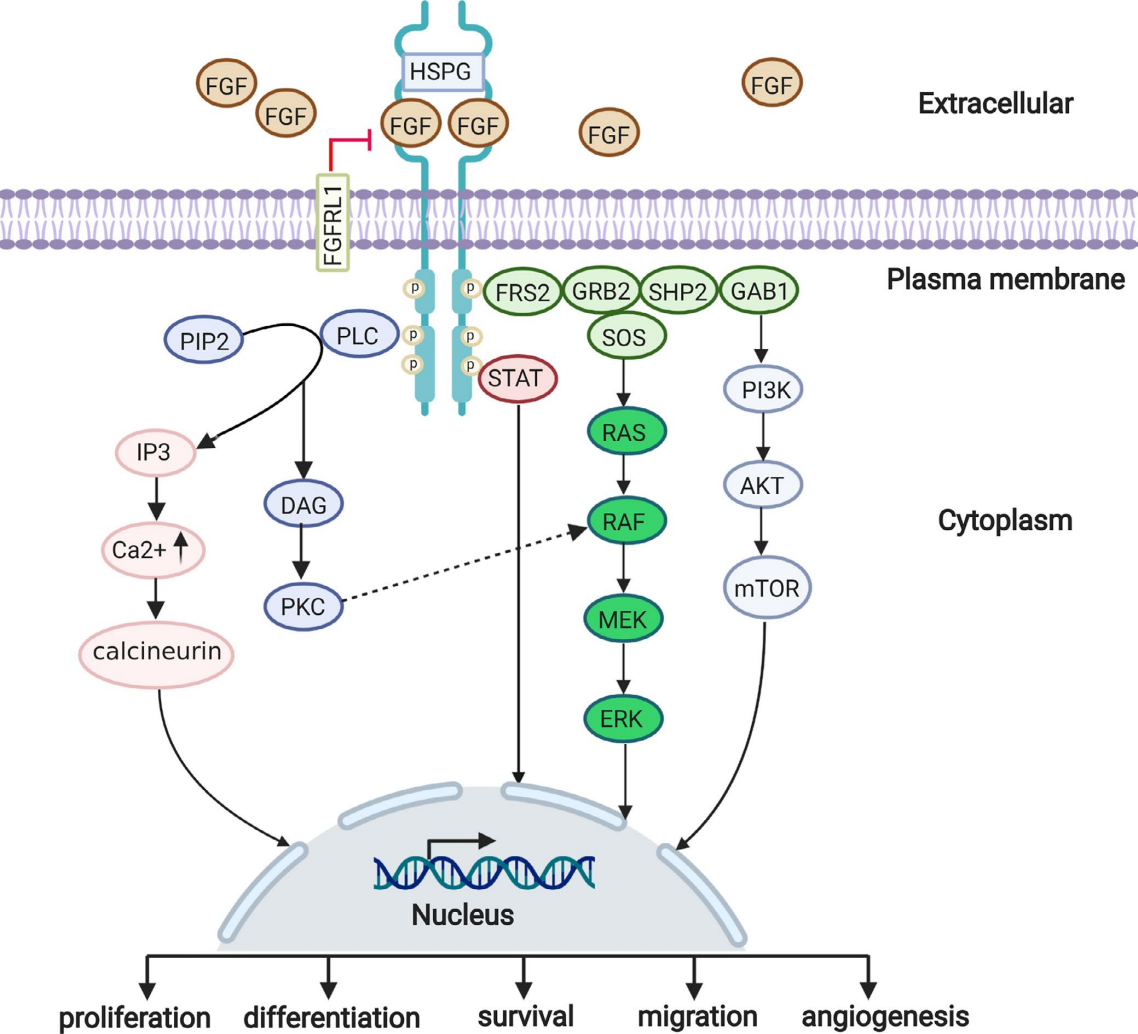
FGF‐FGFR signalling pathway. The binding of FGFs stimulates FGFRs dimerization, resulting in cellular proliferation, differentiation, survival, migration and angiogenesis mainly through Ras‐Raf‐MAPK, PI3K‐AKT, PLCγ and STATs pathways. (See the manuscript for more details) (Created with BioRender.com)

The binding of FGFs drives the dimerization of FGFRs to stimulate the activation of four major intracellular signalling pathways: Ras‐Raf‐MAPK,^26^ PI3K‐AKT,^27^ PLCγ^28^ and STATs.^29^ (Figure 1) Phospho‐FGFR phosphorylates the docking proteins FGFR substrate 2 (FRS2) and FGFR substrate 3 (FRS3). The activated FRS2 binds to growth factor receptor‐bound 2 (GRB2) and tyrosine phosphatase SHP2 proteins. Subsequently, GRB2 recruits SOS and GAB1 to activate the RAS‐MAPK and PI3K‐AKT pathways, respectively.^26^, ^27^ Phosphorylation of Y766 is linked to the initiation of the phospholipase C (PLC‐γ) pathway. Activated PLC‐γ catalyses the hydrolysis of phosphatidylinositol 4,5‐bisphosphate (PIP2) to generate inositol triphosphate (IP3) and diacylglycerol (DAG). IP3 production elevates the level of intracellular calcium ion while DAG stimulates protein kinase C (PKC).^28^ The STAT pathway is triggered by Y677 phosphorylation.^29^

### 2.5 Roles of FGF‐FGFR signalling in physiology

Through triggering downstream signalling pathways, the FGF‐FGFR signalling participates in various vital physiological processes.^15^, ^30^ By regulating key cell behaviours, such as proliferation, differentiation and survival, the FGF‐FGFR signalling pathway can mediate the development of multicellular organisms to ensure proper morphogenesis in the whole development process and also can regulate angiogenesis and wound repair in adults.^31^ Besides, endocrine FGFs can regulate bile acid metabolism in the liver, lipid metabolism in the white adipose tissue, and phosphate and vitamin D levels in serum.^15^ In contrast, intracellular FGFs, independent of FGFRs, exert their biological activity in their original cells via interaction with cytoplasmic domains of ion‐gated sodium channels and mainly play roles in neuronal functions in the postnatal stages.^32^

### 2.6 FGF‐FGFR signalling in cancer

FGFRs are not constitutively active in non‐malignant cells. The oncogenic role of FGF‐FGFR signalling in driving cancer cell proliferation, survival, migration and invasion is mediated by the upregulation of FGF, FGFR genetic alterations, angiogenesis and immune evasion in the tumour microenvironment.^5^

### 2.7 FGFR genetic alterations

An analysis of 4,853 solid tumours by the next‐generation sequencing technique demonstrated FGFR aberrations in 7.1% of cancers. Among them, gene amplification, gene mutations and gene rearrangement accounted for 66%, 26% and 8%, respectively. FGFR1 had the most common alterations (49%), followed by FGFR3 (23%) and FGFR2 (19%), with FGFR4 owning the least alterations (7%).^33^

#### 2.7.1 Gene amplification

Deregulated gene transcription or amplification can lead to elevated FGFR levels, which can activate FGF‐FGFR signalling in a ligand‐independent manner. The amplification of FGFR1 and FGFR2 is more frequent than that of FGFR3 and FGFR4 (Table 1).^34^

**TABLE 1:** FGFR genetic amplification or overexpression in human cancers.[Correction added on 01 April 2021, after first online publication: cholangiocarcinoma tumor has been moved from FGFR3 to FGFR4 in Table 1.]

Gene	Cancer type	Frequency (%)	Reference
FGFR1	Squamous cell lung cancer	5.1‐41.5	35
	Lung adenocarcinomas	0‐14.7	35
	Small‐cell lung cancer	0‐7.8	35
	Myxofibrosarcoma	20	5
	Osteosarcoma	9	44
	Rhabdomyosarcoma	3	209
	Undifferentiated pleomorphic sarcomas	7	210
	Hormone receptor‐positive breast cancer	15	211
	Triple‐negative breast cancer	5	212
	Head and neck squamous cell carcinoma	9.3‐17.4	45
	Prostate cancer	16	213
	Ovary cancer	5	33
	Bladder cancer	2	34
	Oesophageal cancer	9	214
	Gastric cancer	2	215
	Colorectal cancer	6	216
	Pancreatic cancer	1	217
FGFR2	Gastric cancer	5–10	50
	Intrahepatic cholangiocarcinoma	12	218
	Overall breast cancer	2	219
	Triple‐negative breast cancer	4	219
FGFR3	Head and neck squamous cell carcinoma	3	33
	Oral squamous cell carcinoma	48	51
	Oropharyngeal squamous cell carcinoma	59	51
	Oligometastatic colorectal cancers	15	53
	Urothelial cancers	3	33
FGFR4	Cholangiocarcinoma tumour	50	54
	Liver cancer	31.60	55

Amplification of the FGFR1 gene is the most common in all types of FGFR gene alterations. It has been described in a plethora of human tumour types with different ratios.^33^ Recent studies described that the rate of FGFR1 amplification was significantly higher in squamous cell lung cancer (SqCLC) and Asians, and FGFR1 amplification may be a potential new therapeutic target for individual patients with specific lung cancer subtypes such as EGFR TKI for Asian patients with lung adenocarcinoma.^35^ FGFR1‐amplified lung cancer models respond to FGFR inhibitors in preclinical studies in both non‐small cell lung cancer (NSCLC) and small‐cell lung cancer (SCLC), especially in SqCLC, with 9.3% in stage I, 22% in stage II, and 19% in stage IV with brain metastasis.^36^ However, several phase II clinical trials found its limited activity in FGFR1‐amplified lung cancer patients with an acceptable safety profile.^37^ The relationship between amplification of FGFR1 and prognosis is still in doubt in NSCLC. Maybe, it is because of the FGFR1 amplicon co‐amplified with other genes that could contribute to carcinogenesis.^38^ In HR (+)/HER2 (‐) breast cancers, increased expression of FGFR1 was found in hormone‐resistant breast cancer and in patients who received CDK4/6 inhibitors, and these patients can receive 19% of the objective response rate (ORR) treated by lucitanib.^39^, ^40^ Combination of FGFR1 and CDK4/6 inhibitors can effectively suppress FGFR1 and aromatase activities and prolong median progression‐free survival (PFS) by 5.4 months in FGFR1 amplified group in a phase II clinical trial.^39^ FGFR1 amplification is an independent biomarker of a poor prognosis in patients with ER (+) breast cancer.^41^ Moreover, FGFR1 and/or FGF3 gene amplification is associated with resistance to HER2 targeted therapy, a shorter PFS survival and a lower pathological complete response (CR) in HER2 (+) early breast cancer treated with neoadjuvant anti‐HER2 therapy.^42^ What is more, allelic loss and amplification of FGFR1 can predict chemo‐ and radiotherapy response in breast cancer.^43^ FGFR1 amplification correlating with inadequate response to traditional treatments also happens in osteosarcoma,^44^ and the expression of FGFR1 is associated with worse disease‐free survival (DFS) and poor overall survival (OS) in head and neck squamous cell carcinoma (HNSCC),^45^ oesophageal cancer^46^ and colorectal cancer (CRC).^47^

Amplification of FGFR2 is less frequent than that of FGFR1 and mainly focuses on FGFR2, with few other genes co‐amplified. FGFR2 amplification exists in several cancers. Among them, gastric cancer is the most thoroughly studied.^48^ High‐level FGFR2 amplification is associated with the lower response, resistance to chemotherapy, shorter PFS and shorter OS in gastric cancers. Animal experiments show retarded tumour growth in FGFR2‐amplified gastric cancer treated with FGFR inhibitors.^49^ A phase III study demonstrated an ORR of 19% in late‐line gastric cancer with FGFR2 inhibitor. The addition of FGFR2 inhibitor to modified FOLFOX6 for advanced FGFR2‐positive gastroesophageal cancer is ongoing.^50^

It is reported relatively less in amplification of FGFR3 and FGFR4. However, FGFR3 is overexpressed in around 50% of oral and oropharyngeal squamous cell carcinoma.^51^ FGFR3 amplification is also found in HNSCC, urothelial cancers and CRC.^33^ High expression of FGFR3 is concerned with poor prognosis in papillary bladder cancers and oligometastatic CRC.^52^, ^53^ Amplification in FGFR4 occurs in cell lines of rhabdomyosarcoma, prostate and liver cancers. 50% of cholangiocarcinoma and 31.6% of liver cancer patients displayed FGFR4 overexpression concerning cancer initiation and progression.^54^, ^55^

#### 2.7.2 Gene mutations

Both somatic activating mutations and germline single‐nucleotide polymorphisms (SNPs) in FGFRs have been reported to associate with cancer incidence. The research conducted by Greenman et al found more than 1,000 somatic mutations in the coding exons of 518 kinase genes from 210 different cancers, whereas the FGF‐FGFR signalling pathway was the most commonly mutated genes.^56^ Mutations in FGFRs are variable, occurring in the extracellular fragment, TM domain or kinase domain. Somatic activating mutations of FGFR2 and FGFR3 are more common than those of FGFR1.^37^

N546K mutation in the kinase domain of FGFR1 is the most common reported subtype among all the types of FGFR1 mutations. It has been found in Ewing sarcoma, glioblastomas, gastrointestinal stromal tumours and pheochromocytomas.^57^, ^58^, ^59^ Other mutations in FGFR1, such as K565E, have also been reported in glioblastoma.^60^ RNA interference of FGFR1 expression in Ewing sarcoma lines blocked proliferation and completely suppressed xenograft tumour growth.^57^

Unlike the mutations in FGFR1, the most common mutations of FGFR2 are S252w and P253R occurring in the extracellular fragment, while K650E/M/N and N549K in FGFR2 are also found in the A‐loop. FGFR2 mutations are found in up to 12% of endometrial carcinomas, 10% of gastric tumours, approximately 4% of NSCLCs and <2% of urothelial cancers.^61^ FGFR2 mutation is an independent prognostic factor in endometrioid endometrial cancer through disrupting cell polarity to enhance migration and invasion.^62^ However, a phase II study failed to prove that the proportion of patients who were progression‐free at 18 weeks was higher in advanced or metastatic endometrial cancer with FGFR mutations than in FGFR‐non‐mutated endometrial cancer when treated by dovitinib, a TK inhibitor (TKI) of FGFRs, VEGFRs, PDGFR‐beta and c‐KIT after first‐line chemotherapy.^63^

FGFR3 mutations commonly occur in the extracellular (R248C, S249C) and TM (G370C, Y373C) domains of the receptor, which are found to have the ability to stimulate proliferation in cell lines and lead to the transformation of fibroblasts into tumour cells.^33^ 75% of muscle‐non‐invasive bladder cancers (MNIBC) have mutations in FGFR3, while the proportion is around 15% in muscle‐invasive bladder cancers (MIBC).^64^ Mutations in FGFR3 indicate a better prognosis in MNIBC, a better response to neoadjuvant chemotherapy in MIBC and disease occurrence or recurrence in bladder cancers.^65^ At the same time, FGFR3 S249C mutation in urinary cell‐free DNA could predict early‐stage (≤pT1) of upper muscle‐invasive urothelial carcinoma with 100% positive predictive value.^66^ Besides, FGFR3 mutations also occur in cervical, vulvar squamous cell carcinoma and breast cancer.^67^, ^68^, ^69^

The kinase domain mutations of FGFR4 (V550E/L and N535D/K) were described in 7% of rhabdomyosarcoma, leading to tumour growth in vivo and drug resistance to all type I and some type II inhibitors in patients.^70^ Besides, variant rs351855‐G/A can lead to germline FGFR4 G388R substitution, subsequently expose a membrane‐proximal STAT3‐binding site and trigger STAT3 signalling cascade, which can accelerate cancer progression and also contribute to tumour‐extrinsic immune evasion.^71^ FGFR4 G388R substitution is correlated with poor survival in resected colon cancer and lung cancer.^72^, ^73^

#### 2.7.3 Gene fusions

Different gene fusions of FGFRs can lead to variable expression of fusion proteins, which contain a transcription factor and TKs with the ability to induce ligand‐independent receptor dimerization and oncogenic effects. Gene fusions referred to chromosomal translocations in haematological malignancies and chromosomal rearrangements in solid tumours. Compared to fusions in FGFR1‐3, FGFR4 fusions are rarely reported.^37^

Gene fusions with FGFR1 have been found in myeloid/lymphoid neoplasm, lung cancer, papillary thyroid carcinoma, low‐grade gliomas and phosphaturic mesenchymal tumour.^74^, ^75^, ^76^ Among them, FGFR1‐translocated myeloid and lymphoid neoplasms are the most frequently reported, for example, TFG‐FGFR1, BCR‐FGFR1, CNTRL‐FGFR1, ZNF198:FGFR1/ZMYM2‐FGFR1, CEP110‐FGFR1 and FGFR1OP2‐FGFR1 and even achieved complete remission in some patients when treated by FGFR inhibitor.^77^

FGFR2 fusions occur in around 10%‐20% of patients with intrahepatic cholangiocarcinoma. The major fusion partners of FGFR2 are PPHLN1, AHCYL1, BICC1 and TACC3, which bring the probability of targeted therapy for the patients who have FGFR2 rearrangements.^78^ Several FGFR inhibitors have been tested in phase I or II clinical trial and finally, pemigatinib, an FGFR1‐3 inhibitor, received accelerated approval in April 2020 by the FDA for the treatment of patients with previously treated, unresectable, locally advanced or metastatic cholangiocarcinoma with an FGFR2 fusion or other rearrangements based on FIGHT‐202 phase II clinical trial, in which 35.5% of patients with FGFR2 fusions or rearrangements achieved an objective response.^7^ Interestingly, FGFR2 fusions also have been found in breast, prostate and thyroid cancer.^33^

In addition to the presence of FGFR3 amplification and mutations in urothelial carcinoma, FGFR2/3 fusions have also been detected. FGFR3‐TACC3 is an oncogene and has been found in urothelial carcinoma, glioblastoma, lung adenocarcinomas, cervical cancer, triple‐negative breast cancer (TNBC) and oesophageal cancer.^79^, ^80^, ^81^, ^82^, ^83^, ^84^ The fused protein can phosphorylate the phosphopeptide PIN4 through activating mitochondria and subsequently promote mitochondrial respiration and tumour growth. Other researchers found the fused protein can trigger the MAPK‐ERK and JAK‐STAT signalling pathways.^80^, ^84^ Last year, erdafitinib was granted accelerated approval by the FDA for FGFR‐altered urothelial carcinoma progressing on platinum‐based chemotherapy, with an ORR of 40%, a median PFS of 5.5 months and a median OS of 13.8 months in an open‐label, single‐armed BLC2001 phase II trial.^6^

### 2.8 Upregulation of FGFs

Genetic alterations mentioned above mainly lead to constitutive receptor activation and ligand‐independent signalling. However, the ligand‐dependent signalling triggered by FGFs also contributes to the pathogenesis of cancer. The increased amount of FGFs comes from the secretion of cancer cells and (or) the surrounding stromal cells, also referred to as autocrine and paracrine ligand signalling.^37^ Multiple FGFs have been found elevated in different kinds of tumours, such as FGF2 in leukaemia, lung and breast cancer, FGF8 in breast and prostate cancer, FGF10 in lung cancer, FGF19 in hepatocellular carcinoma (HCC) and TNBC.^85^ Interestingly, different kinds of FGFs can be found in one type of tumour. FGF3, FGF4 and FGF19 co‐increase has been detected in approximately 15% of TNBC. FGF1, FGF2, FGF6, FGF8, FGF19 and FGF23 are involved in prostate cancer development and progression.^86^

### 2.9 Angiogenic effects

Although FGF‐FGFR signalling plays a significant role in tumour growth, as discussed above, actually FGFs were firstly found as angiogenic factors. FGF1, FGF2, FGF4 and FGF8 are demonstrated to have pro‐angiogenic effects in different models, especially for FGF1 and FGF2, while other members of canonical FGFs have few or controversial data.^87^ The intratumoral levels of FGF2 mRNA or protein do not correlate with intratumoral vascular density in most cases but correlate with the clinical outcome in some types of cancer (eg breast cancer and HCC).^88^ Endothelial cells also express different members of the FGFR family, including FGFR1IIIc, FGFR2‐IIIc and FGFR3IIIc. The FGF‐FGFR signalling exerts potent pro‐angiogenic properties by promoting endothelial cell proliferation, migration, tube formation, protease production and other biological behaviours.^89^ The inhibition of FGF‐FGFR signalling in endothelial cells disintegrates adhesion and tight junctions, looses endothelial cells and finally disassembles the vasculature. Neutralizing FGF2 and FGFRs inhibit neovascularization and tumour growth in vivo models.^90^ Though not required for vascular homeostasis or physiological function, FGF‐FGFR signalling plays a pivotal role in tissue repair and neovascularization following injury, which validates endothelial cell FGFRs as a target for diseases associated with aberrant vascular proliferation.^91^

### 2.10 Targeting FGF‐FGFR signalling in cancer

As the role of FGF‐FGFR signalling in tumourigenesis, a large number of drugs targeting this signalling pathway have been developed. Except for erdafitinib and pemigatinib approved for urothelial carcinoma and cholangiocarcinoma, respectively, as mentioned above, more inhibitors are under preclinical or clinical trials in various FGFR‐altered tumours. According to their action mechanism, they can be divided into several categories: (a) small‐molecule FGFR TKIs, (b) anti‐FGFR antibodies and (c) and FGF ligand traps.^37^

Actually, FGFR TKIs are the most widely used therapeutic approach, which can be classified into different groups according to different criteria. Firstly, the FGFR TKIs may target other growth factor receptors, as the binding pocket of ATP‐competitive FGFRs shares a high degree of homology with other receptor TKs (RTKs) such as VEGFR and PDGFR. Accordingly, they can be divided into multikinase FGFR inhibitors and FGFR‐specific TKIs.^5^ FGFR inhibitors can be further classified into type I, type II and other types of reversible and/or irreversible inhibitors. Type I and type II inhibitors bind to the ATP‐binding pockets of FGFRs in the active DFG‐in and inactive DFG‐out configuration, respectively, while BLU‐554, FGF401 and TAS‐120 bind covalently to their FGFR target and are divided into type VI inhibitors.^92^ Furthermore, according to the interaction between a small molecular inhibitor and the ATP‐bind pocket in the kinase domain, FGFR inhibitors can be covalent (irreversible) or non‐covalent (reversible) inhibitors. Covalent inhibitors, also called irreversible inhibitors, are thought to have a better binding affinity and selectivity.^93^

Though the approval of erdafitinib and pemigatinib brings some hope in targeting the FGF‐FGFR signalling pathway, many early phases of clinical trials have been terminated for limited efficacy or demonstrated minimal clinical benefit without further researches.^94^ Responses to FGFR‐targeted treatments may be hampered by the activation of bypass signalling pathways and the appearance of secondary drug‐resistant FGFR mutations, FGFR amplification without alterations in protein expression, and intratumour heterogeneity.^37^ Combination inhibition of the FGF‐FGFR signalling pathway with other mechanisms, for example, endocrine therapies, immunotherapies and other targeted therapies may have the potential to enhance the antitumour effect of FGFR TKIs, as well as broaden their indications.^37^ Among these methods, VEGF‐VEGFR signalling deserves attention.

## 3 VEGF‐VEGFR SIGNALLING

### 3.1 VEGFs

One hundred years ago, the growth of tumours had already been thought to rely on blood supply. It was not until 1939 that tumour cells were supposed to release a blood vessel growth factor by themselves.^9^ And then, in 1971, Folkman speculated that tumours could be treated through anti‐angiogenesis.^95^

Inspired by these hypotheses, vascular permeability factor (VPF) was found by Senger, and his colleagues in 1983.^96^ Ferrara and co‐workers isolated VEGFA in 1989. What is more, cDNA and protein sequence analyses proved that VPF and VEGFA were the same molecules.^9^

In mammals, the VEGF family consists of five members, VEGFA, B, C, D and placenta growth factor (PLGF), encoded from the same gene and organized in an anti‐parallel fashion to form a dimer.^97^ In particular, VEGF, referred to as VEGFA, is a major regulator of normal and abnormal angiogenesis. Because of alternative splicing, several variants of VEGFA have been detected, mainly VEGFA121, VEGFA165, VEGFA189 and VEGFA206.^98^

The ability to interact with VEGFR co‐receptors and proteolytic processing decide the bioactivities of the VEGFA isoforms.^99^ Lacking the HSPG‐ and neuropilin‐binding domains, VEGFA121 is a diffuse molecule and cannot remain on the cell surface and in the extracellular matrix (ECM). VEGFA165 has two properties: it can be secreted or stored in the vicinity of the producer cell. On the other hand, VEGFA189 and VEGFA206 include HSPG‐ and neuropilin (NRP)‐binding domains and can bind to co‐receptors with greater affinity than VEGFA165. In addition, protease cleavage of VEGFA189 allows the release of an active, freely diffusible VEGFA110. In other words, VEGFA165 is the most active of all subtypes.^99^, ^100^

Hypoxia is the primary inducer of VEGF gene transcription via hypoxia‐inducible factor (HIF). Besides, growth factors, hormones, cytokines and oncogenic mutations can also influence the production of VEGF.^101^

### 3.2 VEGFRs

**FIGURE 2:**
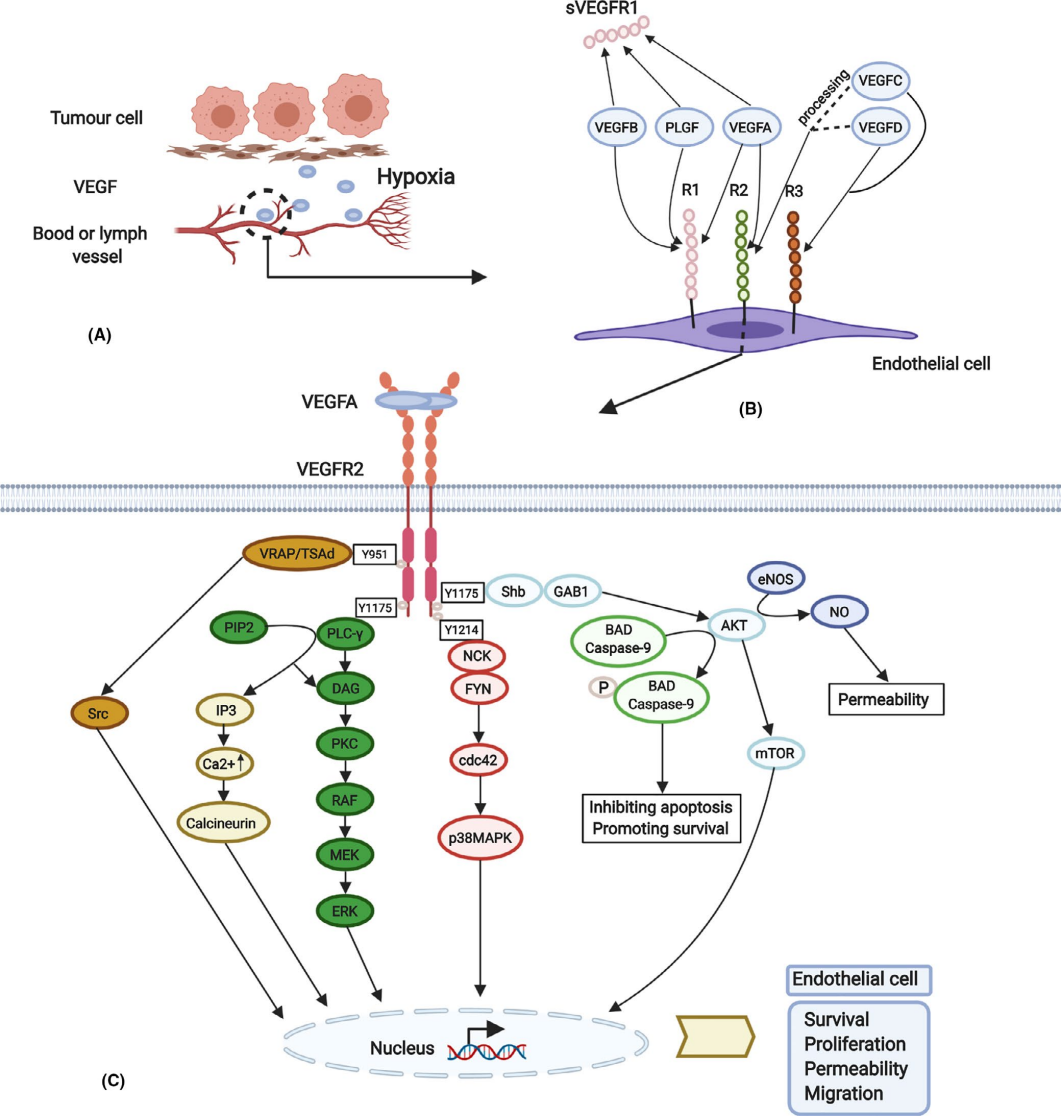
The promotion, composition and intracellular activation of the VEGF‐VEGFR signalling pathway. A, Hypoxia promotes VEGF production. B, Different mammalian VEGFs bind to the three VEGFRs fixedly. C, Binding of VEGFA stimulates VEGFR2 dimerization, resulting in endothelial cellular survival, proliferation, permeability and migration mainly through phosphorylation sites Y1175, Y951 and Y1214. (see the manuscript for more details) (Created with BioRender.com)

These ligands bind in an overlapping pattern to VEGFR1‐3 and have seven Ig‐like domains in the extracellular domain, a single TM region and a split TK domain (Figure 2).^102^ Except for VEGFA121, VEGFA isoforms also interact with the NRP co‐receptors (NRP1 and NRP2), which lack established VEGF‐induced catalytic function but can enhance the function of VEGFR2. VEGFA, B and PLGF bind to VEGFR1, VEGFA binds to VEGFR2, and VEGFC and D bind to VEGFR3. Proteolytic processing of the human VEGFC and D allows for binding to VEGFR2. The Ig‐like domains 2 and 3 are the binding area.^103^ However, VEGFR2 is the central signalling receptor for VEGFA and VEGFR1 acts as a decoy receptor, sequestering VEGFA and thus regulating VEGFR2 activity.^104^

### 3.3 Intracellular signal transduction

Among the downstream pathways of VEGFR1‐3, VEGFR2 is the most thoroughly studied (Figure 2). Y1175, Y951 and Y1214 are the three major VEGFA‐dependent phosphorylation sites in VEGFR2.^105^ Phosphorylated Y1175 (pY1175) can bind PLC‐γ, the adaptor protein Shb and the adaptor protein Sck, further promoting the cascade signalling.^106^ Similar to the FGF‐FGFR pathway, activated PLC‐γ promotes PIP2 to produce IP3 and DAG. Different from the FGF‐FGFR pathway, PKC can initiate the Raf‐MEK‐ERK pathway, independent of Ras, which is central to the proliferation of endothelial cells. Besides, pY1175 can recruit GAB1 to active the PI3K‐AKT pathway. Subsequently, AKT directly phosphorylates two apoptotic proteins, Bcl‐2 associated death promoter (BAD) and caspase‐9, inhibiting their apoptotic activity and promoting cell survival.^107^ In addition, AKT can stimulate the activity of endothelial nitric oxide synthase (eNOS) and further mediate the generation of nitric oxide (NO) to lead to VEGF‐induced permeability.^102^ Phosphorylated Y951 promotes the formation of complexes between Src through the adaptor protein VRAP/TSAd, resulting in the opening of inter‐endothelial junctions, critical for cytoskeletal reorganization and migration.^108^ Phosphorylated Y1214 associates with VEGF‐induced actin remodelling via binding the adaptor protein Nck. Nck interacts with the Src family kinase Fyn leading to activation of Cdc42 and p38 MAPK.^103^

VEGFR1 functions as a decoy receptor that binds its ligands and prevents VEGF binding to VEGFR2, while it is also proved to trigger PI3K and MAPK pathways in transfected cell lines.^103^ VEGFR3 activates the PI3K‐AKT/PKB pathway and the ERK1/2 in a PKC‐dependent manner, just as VEGFR2. Besides, VEGR3 can also trigger the activity of STAT3 and STAT5.^109^

### 3.4 Roles of VEGF‐VEGFR signalling in physiology

VEGFR1 is expressed on haematopoietic stem cells, monocytes, macrophages and vascular endothelial cells. Accordingly, it is required to recruit haematopoietic stem cells and for the migration of monocytes and macrophages. VEGFR1‐/‐ mice die at E8.5‐9.5 due to disorganization induced by excessive proliferation of angioblasts.^102^ VEGFR2 is critical for vascular endothelial cell development, which concerns vasculogenesis during embryogenesis and angiogenesis in the adult, as it is mainly expressed on vascular endothelial cells.^110^ Lacking one of the two VEGF alleles or VEGFR‐2‐/‐ can lead to early embryonic lethality due to defective vascular development.^102^ In adults, skeletal growth and repetitive functions are closely related to angiogenesis. VEGFR2 can also express on neuronal cells, megakaryocytes and haematopoietic stem cells,^107^ while VEGFR‐3 is almost restricted to lymphatic endothelial cells and correspondingly regulates its development.^8^

### 3.5 VEGF‐VEGFR signalling in cancer

A tumour needs angiogenesis to ensure oxygen and nutrients for its growth. VEGF secreted by tumour cells and their microenvironment, binding to VEGFR2, plays the most crucial role in vascular permeability and neo‐angiogenesis.^95^ What is more, the capillary and vascular network facilitates tumour cells to metastasis and spread to distant organs. Studies also found that VEGF can induce immunosuppression by inhibiting cytotoxic T lymphocyte and dendritic cell development and increasing the recruitment and proliferation of immunosuppressive cells, such as Treg cells, MDSCs, and pro‐tumour, M2‐like TAMs, resulting in tumour growth by allowing the escape of tumours from the host immune system.^111^ The expression of VEGFA and VEGFR2 mRNA is upregulated in most human tumours, correlating with tumour recurrence, metastasis and poor prognosis.^94^ Though VEGFR1 acts as a decoy receptor most of the time, it can also be expressed on cancer cells, where it exerts a role in tumour cell survival and growth. Furthermore, the signalling triggered by VEGFR1 can induce the formation of matrix metalloproteinase‐9 and facilitate tumour metastases through recruiting monocytes and macrophages.^112^ Besides, VEGFR‐3 signalling also deserves attention. Malignant cells can escape from their resident tumour and traffic along the lymphatic tracts to the lymph nodes. After entering into the circulation, they can form a malignant mass on other sites in the body.^113^

### 3.6 Targeting VEGF‐VEGFR signalling in cancer

In 1993, the finding that a monoclonal antibody can target and neutralize VEGFA and inhibit tumour growth in the xenograft model led to the translational possibility for targeting VEGF‐VEGFR signalling.^114^ These agents can be divided into two broad classes: agents targeting the VEGF ligand and agents designed to target the cell surface receptor.^115^

As bevacizumab (Avastin) was demonstrated to improve the response rate and survival of patients with CRC combined with chemotherapy, it became the first approved anti‐VEGF monoclonal antibody by the FDA in 2004.^116^ Since then, bevacizumab, in combination with standard treatments, has gained more and more indications.^117^, ^118^, ^119^

Many small‐molecule inhibitors of the VEGFRs have been developed to target the ATP‐binding site of the RTKs, resulting in the blockade of downstream intracellular signalling pathways. Monotherapy with the VEGFR TKIs has mainly proved efficacious in metastatic renal cell carcinoma (RCC), advanced HCC and thyroid cancer.^120^, ^121^, ^122^

Besides, a soluble VEGF decoy receptor (Aflibercept, Zaltrap) neutralizing VEGFA, VEGFB and PLGF was approved in 2012 by the FDA to treat metastatic CRC.^123^ Besides, ramucirumab (Cyramza), a fully human monoclonal antibody that inhibits VEGFR2, has been approved for use in various solid tumours.^124^

The treatment with those anti‐angiogenic drugs has shown benefit in some patients with advanced cancers, but more drugs lead only to mild clinical benefits. The primary or acquired resistance mediated by both tumour cells and stromal cells may explain the minimal benefits.^9^ The resistant mechanisms derived from anti‐angiogenic drugs are different from the inhibitors of well‐defined oncogenic pathways. So far, there is no definitive evidence of pre‐existing or acquired mutations in VEGFA or its signalling pathway.^125^ Upregulation of alternative angiogenic factors, including FGF, plays a vital role in the induction of resistance to VEGF/VEGFR inhibitors.^126^

## 4 TARGETING FGF‐FGFR AND VEGF‐VEGFR SIGNALLING IN CANCER

### 4.1 Combination rationale

The prominent roles of the FGF‐FGFR and VEGF‐VEGFR signalling in tumour cells and angiogenesis have been described in detail earlier in this article. Except for those, other mechanisms, especially combined or interactive mechanisms, deserve further exploration.

As mentioned above, FGF‐FGFR and VEGF‐VEGFR signalling pathways can promote angiogenesis. Interestingly, both FGF and VEGF can be stored on the ECM‐associated HSPGs, and studies have shown that these two pathways have synergistic effects as inducers of angiogenesis.^127^ Researchers have found the combination of FGF‐1 and VEGF induced a more significant angiogenic effect than the additive effects of FGF‐1 or VEGF alone in vitro quantitative fibrin‐based 3‐dimensional angiogenesis system.^128^ Besides, FGFR regulated the secretion of VEGF in a MAPK‐dependent manner, and VEGF, in turn, upregulates the expression of FGF. FGF can also induce the VEGFR2 expression in an ERK1/2‐dependent pathway, and the expression of VEGFR2 rapidly declines without this interaction.^129^ What’ more, neutralizing the VEGF antibody reduced FGF‐driven angiogenesis, implying that VEGF is a crucial mediator that existed downstream of FGF.^127^ It is not surprising that targeting both VEGFR and FGFR resulted in synergistic anti‐angiogenic effects in vivo. A similar synergism is found in lymphangiogenesis, and inhibition of it by dual FGFR/VEGFR inhibitor could prevent metastasis easier.^130^

In addition, upregulation of FGF expression, expressed by pericytes, has been described as a significant mechanism in resistance to anti‐VEGF/VEGFR therapy.^131^ In patients with metastatic RCC who progressed after or were intolerant to sorafenib or sunitinib, dual FGFR and VEGFR inhibitors, including anlotinib, dovitinib and lenvatinib with promising results in phase I or II clinical trials bring them another chance to overcome resistance.^132^, ^133^, ^134^ Lenvatinib and nintedanib also offer opportunities for patients with HCC who progressed on sorafenib treatment.^135^, ^136^

The roles of VEGF‐VEGFR signalling in suppressing tumour immunity have been discussed above. Coincidentally, FGF‐FGFR signalling has similar effects on immune evasion. FGF2 and activation of FGFR1 regulate immunity in the tumour microenvironment by affecting macrophage programming.^137^ VEGF/VEGFR, FGF/FGFR and FGFR/VEGFR inhibitors can invert the TME from immunologically ‘cold’ tumours into ‘hot’ tumours through immune‐supportive effects by decreasing immunosuppressive cells and enhancing infiltration of mature dendritic cells and cytotoxic T lymphocytes.^138^, ^139^, ^140^

**FIGURE 3:**
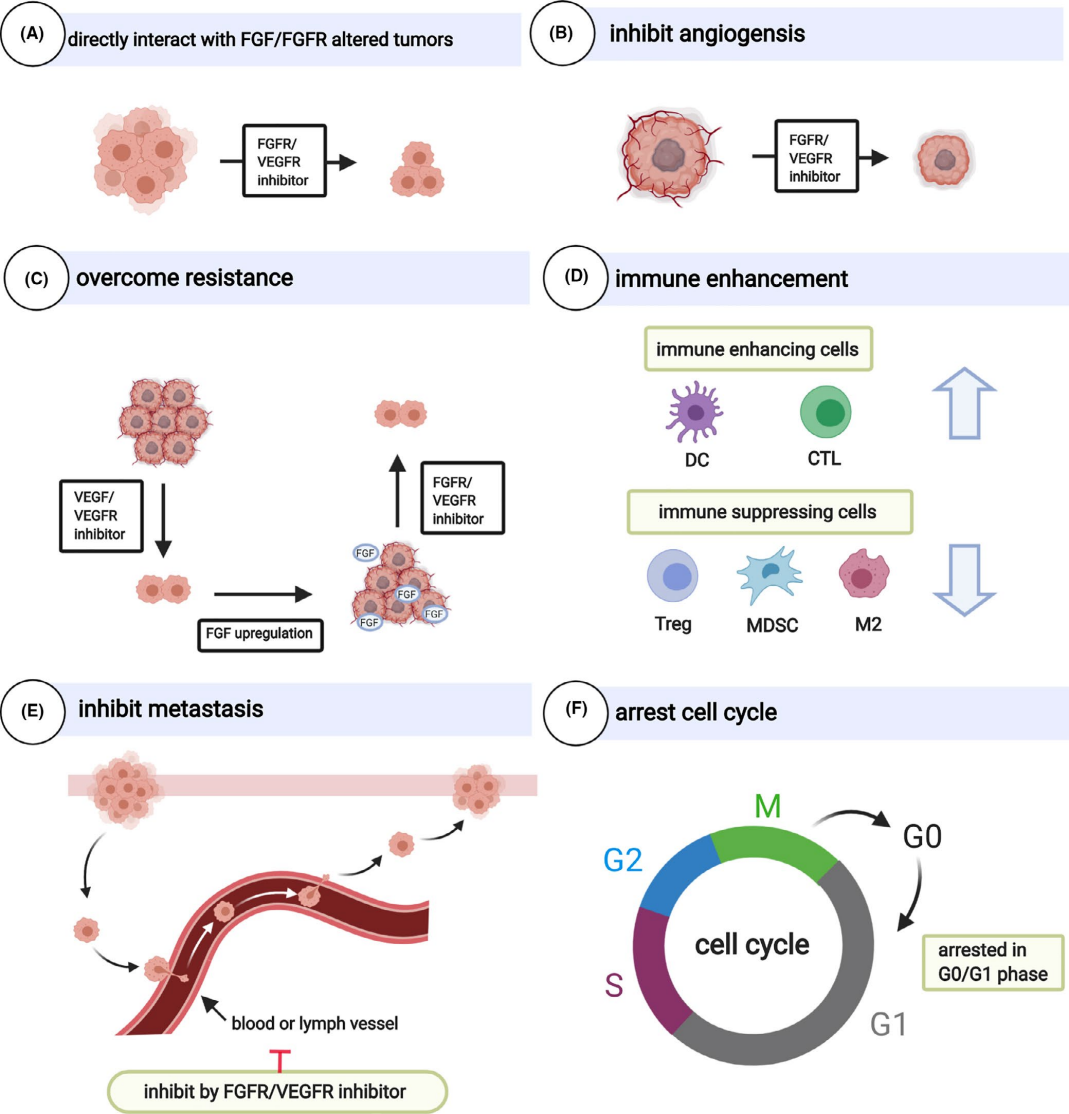
Antitumour mechanisms of FGFR/VEGFR inhibitors. (Created with BioRender.com)

The FGFR/VEGFR inhibitors are also reported to arrest the cell cycle in the G0/G1 phase and cause tumour cell apoptosis.^141^ In general, the dual blockade of FGF‐FGFR and VEGF‐VEGFR signalling cascade is reasonable due to the mechanisms mentioned above (Figure 3). Small‐molecule FGFR/VEGFR inhibitors are preferable because of convenience and economy and are well studied.

### 4.2 Small‐molecule FGFR/VEGFR inhibitors

The small molecular drugs that inhibit FGFR and VEGFR are divided into selective and non‐selective FGFR/VEGFR TKIs according to whether the value of IC50 of inhibitory activity to other kinases is <10 nM.^5^

### 4.3 Non‐selective FGFR/VEGFR TKIs

The values of IC50 and critical clinical trials of multi‐TKIs are listed in Tables 2 and 3, respectively. The details of these drugs will be discussed below.

**TABLE 2:** Classification and specificities of small‐molecule FGFR/VEGFR TKIs

Agent	FGFR1	FGFR2	FGFR3	FGFR4	VEGFR1(Flt‐1)	VEGFR2(Flk‐1)	VEGFR3(Flt4)	Other targets with IC50 < 10 nM	Refs
Non‐selective FGFR/VEGFR inhibitors									
Anlotinib	11.7	NR	NR	NR	82.6	5.6	NR	PDGFR‐β	220
BIBF1000	43	NR	52	NR	40	28	142	PDGFR‐α	221
Derazantinib(ARQ 087)	4.5	1.8	4.5	34	11	21	31	CSF1R, DDR2, KIT, PDGFRs and RET	222
Dovitinib (TKI258)	8	40	9	NR	10	13	8	FLT3, KIT	149
E7090	0.71	0.5	1.2	120	4.9	NR	16	DDR2, RET	155
Lenvatinib (E7080)	61	27	52	43	22	4	5.2	RET and VEGFR1/3	223
Lucitanib (E3810 or AL3810)	18	83	238	>1,000	7	25	10	CSF1R	163
Nintedanib(BIBF1120)	69	37	108	421	34	21	13	CSF1R, KIT, RET	224
Ponatinib (AP24534)	2	2	18	8	NR	1.5	NR	ABL, CSF1R, PDGFRs, RET	225
SOMCL‐085	1.8	1.9	6.9	319.9	5.6	1.2	NR	PDGFR‐β	226
*Selective FGFR/VEGFR inhibitors*									
AZD4547	0.2	2.5	1.8	165	NR	24	NR	—	182
ASP5878	<1	1	1	4	NR	25	NR	—	186
Brivanib (BMS‐540215)	148	NR	NR	NR	380	25	NR	—	227
Erdafitinib(JNJ‐42756493)	1.2	2.5	3	5.7	NR	36.8	NR	—	228
LY2874455	2.8	2.6	6.4	6	NR	7	NR	—	201
ODM‐203	11	16	6	35	26	9	5	—	141
SOMCL‐286	1	4.5	10.6	>1000	79.3%@10 nM	2.9	NR	—	202

**TABLE 3:** Summary of published clinical trials of FGFR/VEGFR TKIs

DRUG(company)	Tumour	Phase	Clinical trial identifier	Sample	Treatment	Comments	Ref
Non‐selective FGFR/VEGFR inhibitors							
Anlotinib(AL3818)(Chia‐tai Tianqing)	Refractory metastatic STS progressed after anthracycline‐based chemotherapy, naïve from angiogenesis inhibitor	II	NCT01878448	166	Anlotinib	Positive	145
	Advanced or metastatic medullary thyroid carcinoma	II	NCT01874873	54	Anlotinib	Positive	144
	Third‐line therapy for refractory advanced NSCLC	II	ALTER 0302	117	Anlotinib vs placebo	Positive	229
	Second‐line therapy for metastatic RCC progressed after or were intolerant to sorafenib or sunitinib	II	NCT02072044	42	Anlotinib	Positive	132
	First‐line therapy for metastatic RCC	II	NCT02072031	133	Anlotinib vs sunitinib	positive	230
	Third‐line or further therapy for advanced NSCLC	III	NCT02388919‐ALTER 0303	439	Anlotinib vs placebo	Positive	143
Derazantinib(ARQ 087)(Basilea)	Advanced solid tumours	I	NCT01752920	80	Derazantinib	Positive	147
	Advanced or inoperable FGFR2 gene fusion‐positive intrahepatic cholangiocarcinoma	I/II	NCT01752920	29	Derazantinib	Positive	148
Dovitinib(TKI258)(Novartis)	Recurrent glioblastoma	I	NCT01972750	12	Dovitinib	Positive; not associated with the FGFR‐TACC gene fusion	231
	Heavily pre‐treated advanced or metastatic RCC	I	NCT00715182	20	Dovitinib	Positive	133
	VEGF refractory RCC	Ib	NCT01714765	18	Dovitinib + everolimus	Negative	232
	Advanced melanoma	I/II	NCT00303251	47	Dovitinib	An acceptable safety profile; limited clinical benefit	233
	Locally advanced or metastatic thyroid cancer	II	NCT01964144	40	Dovitinib	Positive	234
	Recurrent or metastatic adenoid cystic carcinoma	II	NCT01524692	34	Dovitinib	Negative	235
	Metastatic or unresectable adenoid cystic carcinoma	II	NCT01417143	32	Dovitinib	Positive	236
	Previously treated advanced pleural mesothelioma	II	NCT01769547	12	Dovitinib	Negative; terminated ahead	237
	HER2‐ metastatic breast cancer	II	NCT00958971	81	Dovitinib	Positive	151
	Post‐menopausal patients with HER2‐ and HR + breast cancer progression on or after prior endocrine therapy	II	NCT01528345	97	Fulvestrant ± dovitinib	Positive; promising clinical activity in the FGF pathway–amplified subgroup	41
	Metastatic RCC	II	NCT00715182	67	Dovitinib	Positive; effective and tolerable after treatment with VEGFR TKIs and mTOR inhibitors	238
	Second‐line therapy for progressive FGFR3‐mutated or FGFR3 wild‐type advanced urothelial carcinoma	II	NCT00790426	44	Dovitinib	Negative	239
	BCG‐unresponsive urothelial carcinoma with FGFR3 mutations or overexpression	II	NCT01732107	13	Dovitinib	Negative; pFGFR3 not predict response to dovitinib	152
	Castration‐resistant prostate cancer	II	NCT01741116	44	Dovitinib	Positive; high expression of VEGFR2 predict efficacy	235
	Second‐line therapy for FGFR2 mutated or wild‐type advanced and/or metastatic endometrial cancer	II	NCT01379534	53	Dovitinib	Negative; not reach the prespecified study criteria; treatment effects independent of FGFR2 mutation status	63
	Third‐line therapy for metastatic RCC after failure of anti‐angiogenic therapies	III	NCT01223027	564	Dovitinib vs sorafenib	Negative; not better than sorafenib	153
E7090	Advanced solid tumours refractory to standard therapy, or for whom no appropriate treatment was available	I	NCT02275910	24	E7090	Positive	156
Lenvatinib(E7080)(Eisai)	Advanced solid tumours	I	NCT00280397	27	Lenvatinib	Positive	240
	Advanced solid tumours	I	NCT00121719	82	Lenvatinib	Positive	241
	Chemotherapy‐naïve NSCLC	I	NCT00832819	28	Lenvatinib + carboplatin+paclitaxel	Positive	158
	Advanced thyroid cancer	II	NCT01728623	51	Lenvatinib	Positive	242
	Advanced medullary thyroid cancer	II	NCT00784303	59	Lenvatinib	Positive	243
	First‐line or second‐line therapy for advanced gastric cancer	II	NCT03609359	29	Lenvatinib + pembrolizumab	Positive	159
	Advanced HCC	II	NCT00946153	46	Lenvatinib	Positive	135
	Second‐line therapy for metastatic RCC	II	NCT01136733	153	Lenvatinib + everolimus vs lenvatinib vs everolimus	Positive	134
	Advanced endometrial cancer	II	NCT02501096	53	Lenvatinib + pembrolizumab	Positive	161
	Second‐line therapy for recurrent endometrial cancer	II	NCT01111461	133	Lenvatinib	Positive	244
	Radioiodine refractory differentiated thyroid cancer	III	NCT01321554‐SELECT	392	Lenvatinib vs placebo	Positive	157
	First‐line therapy for unresectable HCC	III	NCT01761266‐REFLECT	954	Lenvatinib vs sorafenib	Positive	160
Lucitanib(E3810 or AL3810)	Second or third‐line therapy for HR+/HER2‐ metastatic breast cancer	II	NCT02053636‐FINESSE	76	Lucitanib	Positive; patients with high FGFR1 amplification or expression might derive greater benefit	39
	Advanced solid tumours	I/IIa	NCT01283945	76	Lucitanib	Positive	165
Nintedanib(BIBF1120)(Boehringer Ingelheim)	Second‐line therapy for adenocarcinoma subtype NSCLC	I	NCT02300298	10	Nintedanib + docetaxel	Positive	245
	Second‐line therapy for advanced NSLCL	I	NCT00979576	18	Nintedanib + pemetrexed	Positive	136
	Adjuvant therapy for post‐menopausal women with breast cancer	I	NCT02619162	19	Nintedanib + letrozole	Positive	42
	Unresectable HCC after sorafenib treatment	I	NCT01594125	30	Nintedanib	Positive	136
	Advanced solid tumours	I	NCT00998296	70	Nintedanib + afatinib	Positive	169
	Third‐line or further therapy for advanced solid tumour	Ib	NCT02835833	18	Nintedanib + bevacizumab	Positive; overcome bevacizumab resistance	170
	Elderly patients with AML unfit for an intensive induction therapy	I	NCT01488344	13	Nintedanib + low‐dose cytarabine	Positive	246
	Recurrent high‐grade gliomas	II	NCT01380782	22	Nintedanib	Negative; not active regardless of prior bevacizumab therapy	174
	Second‐line or third‐line therapy for recurrent glioblastoma multiforme	II	NCT01251484	25	Nintedanib	Negative; terminated ahead	247
	Second‐line therapy for SCLC	II	NCT01441297	22	Nintedanib	Negative; failed to proceed	248
	Advanced, recurrent or metastatic endometrial cancer	II	NCT01225887	32	Nintedanib	Negative	171
	Second‐line therapy for stage IIIB/IV or recurrent NSCLC	III	NCT00805194‐LUME‐Lung 1	1314	Docetaxel ± nintedanib	Positive	249
	Unresectable malignant pleural mesothelioma	III	NCT01907100‐LUME‐Meso	545	Pemetrexed + cisplatin±nintedanib	Negative	172
	Refractory metastatic CRC	III	NCT02149108‐LUME‐Colon 1	765	Nintedanib + BSC vs Placebo + BSC	Negative	173
	Advanced ovarian cancer	III	NCT01015118‐LUME‐Ovar 1	1366	Paclitaxel + carboplatin±nintedanib	Positive	250
Ponatinib(AP24534)(ARIAD)	Japanese patients with CML or Ph + ALL	I/II	NCT01667133	35	Ponatinib	Positive	251
	Heavily pre‐treated CML or Ph + ALL	II	NCT01207440‐PACE	449	Ponatinib	Positive	180
	First‐line therapy for Ph + ALL	II	NCT01424982	37	Ponatinib + chemotherapy	Positive	179
	First‐line therapy for Ph + ALL	II	NCT01424982	76	Ponatinib + hyper‐CVAD	Positive	179
	First‐line therapy for CML in chronic phase	II	NCT01570868	51	Ponatinib	Termination ahead for the increased risk of thromboembolism	252
	First‐line therapy for CML	III	NCT01650805	307	Ponatinib vs imatinib	Cannot be assessed due to termination ahead	181
Selective FGFR/VEGFR inhibitors							
AZD4547(AstraZeneca)	Previously treated stage IV FGFR1‐amplified SqCLC	Ib	NCT00979134	15	AZD4547	Poor correlation between gene amplification and expression, potential genomic modifiers of efficacy, and heterogeneity in 8p11 amplicon	253
	Japanese patients with advanced solid tumours	I	NCT01213160	34	AZD4547	Well tolerated in Japanese patients, with best response of stable disease ≥ 4 weeks	254
	Second‐line therapy for advanced gastric adenocarcinoma with FGFR2 polysomy or gene amplification	II	NCT01457846‐SHINE study	67	AZD4547 vs. paclitaxel	Negative; Considerable intratumour heterogeneity for FGFR2 gene amplification and poor concordance between FGFR2 amplification/polysomy and FGFR2 expression indicates the need for alternative predictive biomarker testing.	184
	Tumours harbouring actionable aberration(s) in FGFR1‐3	II	NCT02465060‐NCI‐MATCH	48	AZD4547	Negative; ORR < 16%	185
	Previously treated patients with FGF pathway‐activated SqCLC	II	NCT02965378‐SWOG S1400D‐Lung‐MAP Substudy	27	AZD4547	Negative; AZD4547 had an acceptable safety profile but minimal activity in FGFR 1/3 amplified cohort.	183
Brivanib(BMS‐540215)(Bristol‐Myers Squibb)	Advanced or metastatic solid tumours	I	NCT00207051	90	Brivanib	Positive	255
	Second‐line therapy for advanced HCC	II	NCT00355238	46	Brivanib	Positive	191
	First‐line therapy for advanced HCC	II	NCT00355238	55	Brivanib	Positive	190
	Persistent or recurrent cervical cancer following at least one prior cytotoxic regimen	II	NCT01267253	28	Brivanib	Positive; terminated ahead due to lack of drug	256
	Advanced HCC who were intolerant to sorafenib or for whom sorafenib failed	III	NCT00825955‐BRISK‐PS	395	Brivanib + bsc vs placebo + bsc	Negative	194
	First‐line therapy for unresectable, advanced HCC	III	NCT00858871‐BRISK‐FL	977	Brivanib vs. placebo	Negative	195
	Adjuvant therapy to transarterial chemoembolization in patients with HCC	III	NCT00908752	502	Brivanib vs placebo	Negative	193
	Metastatic, chemotherapy‐refractory, wild‐type K‐RAS CRC	III	NCT00640471	750	Cetuximab ± brivanib	Negative	192
ASP5878	Solid tumours	I	NCT02038673	86	ASP5878	Positive	188
Erdafitinib(JNJ‐42756493)(Janssen)	Advanced or refractory solid tumours	I	NCT01703481	187	Erdafitinib	Positive	198
	Advanced solid tumours	I	NCT01703481	65	Erdafitinib	Positive	196
	Advanced or refractory solid tumours	I	NCT01962532	19	Erdafitinib	Positive	197
	Locally advanced or metastatic urothelial carcinoma with FGFR3 mutation or FGFR2/3 fusion	II	NCT02365597‐BLC2001	99	Erdafitinib	Positive	6
Ly2874455(Lilly)	Advanced cancer	I	NCT01212107	92	LY2874455	Positive	201

Abbreviations: ALL, acute lymphocytic leukaemia; AML, acute myeloid leukaemia; CML, chronic myeloid leukaemia; CRC, colorectal cancer; HCC, hepatocellular carcinoma; NSCLC, non‐small cell lung cancer; Ph+, philadelphia chromosome‐positive; RCC, renal cell carcinoma; SCLC, small‐cell lung cancer; SqCLC, squamous cell lung cancer; STS, soft‐tissue sarcoma.

#### 4.3.1 Anlotinib

Anlotinib (AL3818) is a multi‐TKI that is designed to inhibit VEGFR1‐3, FGFR1‐4, PDGFRα/β, c‐Kit and Ret and has been approved by the CFDA as a third‐line or beyond therapy for stage IV NSCLC in 2018.^142^ In phase III ALTER‐0303 trial, anlotinib significantly improved median OS from 6.3 months in the placebo group to 9.6 months in the anlotinib group (HR, 0.68; 95%CI, 0.54 to 0.87; *P* =.002) and median PFS from 1.6 months to 5.4 months (HR,0.25; 95%CI, 0.19 to 0.31; *P* =.001).^143^ Besides, anlotinib also showed promising efficacy in patients with metastatic RCC, advanced or metastatic medullary thyroid carcinoma and refractory metastatic soft‐tissue sarcoma (STS) progressed after anthracycline‐based chemotherapy, naïve from angiogenesis inhibitor.^132^, ^144^, ^145^ Interestingly, the incidence of grade 3 or higher side effects is much lower than that of other TKIs.^142^

#### 4.3.2 Derazantinib

Derazantinib (ARQ 087) is an ATP‐competitive inhibitor of FGFR1‐3 and also shows similar activity against FGFR4 and VEGFR2 with the values of IC50 around 30 nM.^93^ It inhibits the growth of FGFR‐addicted cancer cell lines and tumours in preclinical models.^146^ Two phase I clinical trials which have been published demonstrated the safety and efficacy of derazantinib in FGFR2 fusion‐positive intrahepatic cholangiocarcinoma and urothelial cancer with FGFR2 and FGF19 amplification.^147^, ^148^

#### 4.3.3 Dovitinib

Dovitinib (TKI258) is a non‐selective and ATP‐competitive TKI that targets VEGFR1‐3, FGFR1‐3 and PDGFRβ in the nM range of concentration.^149^ Dovitinib has made attempts to target the FGF‐FGFR pathway. In preclinical studies, dovitinib showed the ability to inhibit FGFR1‐ and FGFR2‐amplified, but not FGFR‐normal breast cancer cell lines in vitro and inhibit tumour growth in FGFR1‐amplified breast cancer in vivo.^150^ In phase II clinical trials, dovitinib prolonged DCR and median PFS from 3% and 5.5 months to 25% and 10.9 months in patients with FGFR1‐amplified/HR‐positive breast cancer, respectively.^151^ However, dovitinib did not show clinical benefit in endometrial cancer with FGFR2 mutations, glioblastoma with FGFR3‐TACC3 gene fusion and urothelial carcinoma with FGFR3 mutations or overexpression.^63^, ^152^ Besides, dovitinib failed to show superiority over sorafenib in a phase III study of third‐line therapy for metastatic RCC after failure of anti‐angiogenic therapies and a phase II study of frontline therapy for advanced HCC.^153^, ^154^

#### 4.3.4 E7090

E7090 is an orally non‐selective inhibitor of FGFR1‐3 and has a slightly lower inhibitory activity on VEGFR2.^155^ Phase I clinical trial has demonstrated its safety, but more clinical studies are needed to prove its efficacy in FGFR‐altered tumours.^156^

#### 4.3.5 Lenvatinib

Lenvatinib (E7080) is an oral multikinase inhibitor that targets VEGFR1‐3, FGFR1‐4, RET, c‐kit and PDGFRa, obtained considerable success in clinical trials of different cancer types, including NSCLC, thyroid cancer, gastric cancer, HCC, RCC and endometrial cancer.^134^, ^157^, ^158^, ^159^, ^160^, ^161^ Remarkably, lenvatinib has been approved in differentiated thyroid cancer (DTC), RCC and HCC as a single agent or in combination.^134^, ^157^, ^160^ Lenvatinib broke the situation that sorafenib was the only targeted therapy for radioiodine refractory differentiated thyroid cancer and unresectable HCC in 2015 and 2018, respectively.^157^, ^160^ The median PFS of DTC prolonged from 3.6 months in the placebo group to 18.3 months in the lenvatinib group (HR 0.21; 99% CI: 0.14 to 0.31; *P* <.001) in phase III SELECT trial.^157^ In addition, phase III REFLECT trial demonstrated that median OS with lenvatinib was 13.6 months vs 12.3 months with sorafenib (HR 0.92; 95% CI: 0.79 to 1.06) and median PFS 7.3 months vs 3.6 months (HR 0.64; 95%CI: 0.55 to 0.75; *P* <.001) in unresectable HCC.^160^ What’ more, lenvatinib plus everolimus also showed promising results in a phase II trial, leading to the FDA approval of this combination in advanced RCC following one prior anti‐angiogenic therapy.^134^ Interestingly, many efforts have been made to find the relationship between the outcome and biomarkers based on the REFLECT trial. For example, baseline Ang2, upregulated FGF23 and treatment‐emergent hypertension correlated with improved PFS, and diarrhoea were significantly associated with OS in lenvatinib‐treated patients.^160^ In other words, the factors mentioned above may predict the efficacy of lenvatinib. Nowadays, as lenvatinib was reported to decrease tumour‐associated macrophages and increase infiltration of CD8+ T cells, many clinical trials combining the immune checkpoint inhibitors with lenvatinib are ongoing, and some of them have already got positive results (NCT03609359, NCT02501096).^161^, ^162^

#### 4.3.6 Lucitanib

Lucitanib (E3810 or AL3810) is a reversible, ATP‐competitive TKI that targets FGFR1‐2 and VEGFR1‐3 in the nM range and exerts antitumour activity in multiple preclinical models, including colon, ovarian, renal and thyroid carcinoma and breast cancer.^40^, ^163^, ^164^ Soria JC demonstrated the clinical benefit of lucitanib used in both FGF‐aberrant and angiogenesis‐sensitive populations, with 50% (six of 12) achieved partial response (PR) in FGF‐aberrant breast cancer patients.^165^ Subsequently, the phase II FINESSE study found the ORRs in lucitanib‐treated HR+/HER2‐ metastatic breast cancer with FGFR1 amplification or 11q13 amplification or no amplification were 19%, 0%, and 15%, respectively.^39^ What is more, the following analyses showed that the ORR in patients with high‐level FGFR1 amplification was higher in patients without high‐level FGFR1 amplification (22% vs 9%), indicating that FGFR1 may be a biomarker for FGFR inhibitor therapy.^39^

#### 4.3.7 Nintedanib

Nintedanib (BIBF1120) is a non‐selective FGFR TKI that competitively and reversibly blocks the ATP‐binding pocket of FGFR1‐3, VEGFR1‐3 and PDGFR.^166^ This inhibitor has obtained promising results on different cancers in preclinical studies as a single agent or combination with standard chemotherapies, including lung, prostate, colorectal, pancreatic, ovarian cancer and STS.^166^, ^167^, ^168^ Based on these results, nintedanib has been or is being tried in various tumour types in clinical trials. Most phase I studies have shown nintedanib to be safe and efficacious at 200mg bid,^42^, ^136^, ^169^, ^170^ but it frequently showed limited efficacy in most phase II and III studies.^171^, ^172^, ^173^, ^174^, ^175^ Fortunately, nintedanib was approved by EMA for its second‐line use in combination with docetaxel in patients with lung adenocarcinoma based on the results of the phase III LUME‐Lung 1 study in November 2014.^176^ To get better results, molecular biomarkers concerning FGFR1, FGF23 and VEGFR2 deserve to be considered.^177^

#### 4.3.8 Ponatinib

Ponatinib (AP24534) is a multi‐TKI targeting SRC, ABL, FGFR, PDGFR and VEGFR, while the inhibition of BCR‐ABL is the primary clinical use.^178^ The FDA has approved it to treat patients with heavily pre‐treated CML and Philadelphia chromosome‐positive acute lymphoblastic leukaemia based on the encouraging outcomes of phase II PACE clinical trial.^179^, ^180^ However, the subsequent clinical trials were blocked because of its severe vascular toxicity.^181^ Currently, researchers are trying to discover novel FGFRs inhibitors according to the structure of ponatinib, which have already displayed significant antitumour activities in FGFR1‐amplificated H1581 and FGFR2‐amplificated SNU‐16 xenograft models.^178^

In total, some non‐selective FGFR/VEGFR inhibitors have already got great success in the clinic by simultaneously blocking multiple TKs and concomitantly inhibiting redundant or bypassing pathways. Because of the multiple targets of non‐selective FGFR/VEGFR inhibitors, their antitumour effects are not limited to FGFR‐addicted tumours. On the other hand, they also bring unexpected side effects and weaken the antitumour effects only by inhibiting FGFR and VEGFR.

### 4.4 Selective FGFR/VEGFR TKIs

Nowadays, dual inhibitors of FGFR and VEGFR have been developed. In addition to the basic information listed in Tables 2 and 3, distinct features of these drugs are discussed as follows.

### 4.5 AZD4547

AZD4547 is a selective and reversible TKI of FGFR1‐3 and also shows activity against VEGFR2 at nM concentration with IC50 equal to 24 nM.^182^ Its antitumour effect has been confirmed in some preclinical tumour models, including oesophageal squamous, non‐small‐cell lung, breast, endometrial and colorectal tumours characterized by different kinds of FGFR alterations.^182^ Recently, clinical trials showed that AZD4547 was well tolerated. However, minimal activities were achieved against tumours harbouring actionable aberration(s) in FGFR1‐3, including FGFR1‐amplified SqCLC and gastric adenocarcinoma with FGFR2 polysomy or gene amplification.^183^, ^184^ Two reasons may explain this phenomenon, one is considerable intratumour heterogeneity existed in gene amplification, and the other is gene amplification cannot stand for gene expression.^185^ Taken together, the need for alternative predictive biomarkers is extremely urgent.

### 4.6 ASP5878

ASP5878 is a selective pan‐FGFR inhibitor that exerts its antitumour activity towards tumours with FGFR genetic alterations.^186^ Researchers have demonstrated the role of ASP5878 in FGFR3‐dependent urothelial cancer and FGF‐19‐expressing HCC in the xenograft mouse model.^186^, ^187^ Clinical trials concerning ASP5878 are limited, and only one phase I clinical trial showed that ASP5878 was well tolerated.^188^

#### 4.6.1 Brivanib

Brivanib (BMS‐540215) is a selective dual inhibitor against VEGFR and FGFR, with its main clinical trials focused on HCC.^189^ Brivanib successively received positive results in second‐line and first‐line therapy for advanced HCC in phase II clinical trials,^190^, ^191^ while in phase III clinical trials, brivanib failed without exception.^192^, ^193^, ^194^, ^195^ In second‐line treatment for patients who were intolerant to sorafenib or for whom sorafenib failed, brivanib did not significantly improve OS compared to placebo with median OS 9.4 months in brivanib group vs 8.2 months in placebo (HR,0.89;95.8% CI,0.69 to 1.15; *P* =.3307).^194^ It also did not meet the primary endpoint of OS non‐inferiority for brivanib vs sorafenib (median OS: 9.5 months vs 9.9 months HR, 1.06; 95.8% CI, 0.93 to 1.22) in phase III BRISK‐FL study.^195^ In addition, when brivanib was used as adjuvant therapy to transarterial chemoembolization in unresectable intermediate‐stage HCC, it still did not improve OS.^193^ It also failed to improve OS in wild‐type K‐RAS CRC in combination with cetuximab.^192^

#### 4.6.2 Erdafitinib

Erdafitinib (JNJ‐42756493) is a highly selective and reversible inhibitor of FGFR1‐4 and can inhibit VEGFR2 with IC50 equal to 37 nM.^196^ In phase I clinical trials, it showed clinical benefits in glioblastoma, cholangiocarcinoma, urothelial and endometrial cancer with FGFR mutations or fusions, while ORRs in other tumour types were below 10%.^6^, ^197^, ^198^ In April 2019, erdafitinib received accelerated approval by the FDA to treat patients with FGFR3 mutated or FGFR2/3 fusion‐positive advanced or metastatic urothelial carcinoma after at least one prior platinum‐based regimen. The ORR reached 40%, and a median PFS was 5.5 months. At the same time, treatment‐related grade 3 or higher adverse events also happened in nearly half the patients, including hyponatremia, stomatitis and asthenia in phase II BLC2001 clinical trial.^6^ Erdafitinib also received three black‐box warnings by Janssen pharmaceutical company for the risks of ocular disorders, hyperphosphataemia and embryo‐foetal toxicity.^199^

#### 4.6.3 Ly2874455

Ly2874455 is a selective pan‐FGFR inhibitor, with similar values of IC50 in inhibiting FGFR1‐4, which also has inhibitory activity towards VEGFR2 with IC50 equal to 7 nM.^200^ Interestingly, as the inhibition of FGF‐induced Erk phosphorylation by Ly2874455 is much easier than that of VEGF‐mediated target signalling in vivo, LY2874455 can avoid VEGFR2‐mediated hypertension at efficacious doses.^201^ Until now, a phase I clinical trial has published its results demonstrating the excellent tolerability and activity in patients with advanced cancer, especially for patients with gastric cancer and NSCLC.^201^

In addition, some drugs are in the preclinical development stage. For example, ODM‐203 is a selective and equipotent inhibitor of FGFR and VEGFR, which exhibits its equal inhibitory activity towards FGFR and VEGFR families in biochemical assays, cellular assays and in vivo.^141^ SOMCL‐286 starting from the structure of lucitanib is another FGFR and VEGR2 dual inhibitor and showed significant antitumour effects in SNU‐16 xenograft model harbouring aberration in FGFR and VEGFR2.^202^

Overall, only a few selective FGFR/VEGFR inhibitors have entered into phase III clinical trials and subsequently got approved. The clinical effects of these drugs vary with different types of FGFR genetic alterations. The effect of drugs targeting FGFR gene fusion and mutations seems to be better than that of gene amplification, probably mainly because gene amplification does not imply high protein expression. Biomarkers predicting the efficacy of selective FGFR/VEGFR inhibitors deserve explored.

#### 4.6.4 Conclusion and future perspective

FGF‐FGFR signalling can be abnormally triggered by FGF and FGFR alterations.^5^ Besides, both FGF‐FGFR and VEGF‐VEGFR signalling pathways can promote angiogenesis and induce immune evasion.^127^, ^140^ By inhibiting these two signalling cascades, we can both target tumour cells and TME. FGFR/VEGFR dual inhibitors have already received encouraging results in clinical trials, and some of them have already received approval for certain cancers, especially for non‐selective FGFR/VEGFR inhibitors. In order to avoid unexpected side effects of non‐selective FGFR/VEGFR inhibitors and optimize the effect of selective FGFR/VEGFR inhibitors, suitable biomarkers need to be developed to predict the efficacy of selective FGFR/VEGFR inhibitors.^203^, ^204^

Besides, FGF and VEGF induce immunosuppressive microenvironment by inhibiting immune effector cells and recruiting immunosuppressive cells, and FGFR/VEGFR dual inhibitors can revert the TME from immunologically ‘cold’ tumours into ‘hot’ tumours.^205^ At the same time, immune checkpoint inhibitors (ICIs) have been approved in many types of tumours, working through restoring antitumour T‐cell functions.^206^ However, lacking pre‐existing immune cells in TME leads to inadequate response to monotherapy with ICIs. The combination of lenvatinib and pembrolizumab has received accelerated approval in patients with advanced endometrial cancer and is undergoing phase III clinical trial in HCC and RCC (NCT03713593, NCT02811861).^161^, ^207^, ^208^ Combining FGFR/VEGFR dual inhibitors with ICIs is a promising treatment in the future.

## CONFLICT OF INTEREST

The authors declare no competing financial interests.

## AUTHORS’ CONTRIBUTIONS

YW and XW offered direction and guidance of the manuscript. GL and TC drafted the initial manuscript. ZD revised the manuscript. GL and YW illustrated the figures and tables for the manuscript. All authors approved the final manuscript.

## Data Availability

The data that support the findings of this study are available from the corresponding author upon reasonable request.
